# Characteristics and risk of interstitial lung disease in dermatomyositis and polymyositis: a retrospective cohort study in Japan

**DOI:** 10.1038/s41598-023-44092-9

**Published:** 2023-10-11

**Authors:** Qingqing Hu, Kuan-Chih Huang, Choo Hua Goh, Yumi Tsuchiya, Yanfang Liu, Hong Qiu

**Affiliations:** 1 Global Epidemiology, Office of the Chief Medical Officer, Johnson & Johnson, Shanghai, China; 2 Global Epidemiology, Office of the Chief Medical Officer, Johnson & Johnson, Taipei, Taiwan; 3 Global Epidemiology, Office of the Chief Medical Officer, Johnson & Johnson, Singapore, Singapore; 4 Research & Development, Janssen Pharmaceutical K.K., Tokyo, Japan; 5 Global Epidemiology, Office of the Chief Medical Officer, Johnson & Johnson, Titusville, USA

**Keywords:** Idiopathic inflammatory myopathies, Respiratory tract diseases

## Abstract

Dermatomyositis and polymyositis are rare, idiopathic inflammatory myopathies. Interstitial lung disease is one of the most common and potentially severe extra-muscular manifestations of dermatomyositis and polymyositis and is strongly linked to poor prognosis and early mortality. We aimed to characterise the demographic and clinical characteristics, incidence, and treatment of interstitial lung disease in patients with dermatomyositis or polymyositis. We conducted a retrospective cohort study using the Japan Medical Data Center healthcare claims database. Patients in the database with dermatomyositis (International Classification of Disease version 10 M33.0, M33.1, M33.9) or polymyositis (M33.2) from 01-Jan-2011 until 31-Dec-2019 were identified and followed-up for interstitial lung disease (J84.x) until death, dis-enrolment, or study end (31 December 2020). Cumulative risk curves compared interstitial lung disease risk in dermatomyositis versus polymyositis. Risk factors were evaluated by Cox proportional hazard models. There were 886 patients with dermatomyositis and 745 patients with polymyositis included in the cohort analysis. Mean (standard deviation) age at dermatomyositis/polymyositis diagnosis was 46.0 (16.0)/49.7 (13.3) years and 300 (34%)/104 (14%) developed interstitial lung disease during follow-up. The incidence rate of interstitial lung disease per 100 person-years was 18.42 (95% CI 16.42–20.59) for dermatomyositis and 5.39 (95% CI 4.43–6.50) for polymyositis. In the analysis adjusted for sex, age, and comorbidity score, the risk of interstitial lung disease was significantly higher in patients with dermatomyositis than with polymyositis (hazard ratio 2.72, 95% CI 2.18–3.41). The rate diverged markedly between the groups in the first year after diagnosis. Risk factors for interstitial lung disease were older age in dermatomyositis, female sex and rheumatoid arthritis in polymyositis. Glucocorticoids with/without tacrolimus were the most common newly prescribed drugs after the interstitial lung disease diagnosis. In conclusion, the risk of developing interstitial lung disease was significantly higher in patients with dermatomyositis than with polymyositis, and risk factors were different in the 2 patient groups.

## Introduction

Dermatomyositis (DM) and polymyositis (PM) are idiopathic inflammatory myopathies characterised by muscle inflammation and proximal muscles weakness^1,2^. Patients with DM may also develop characteristic skin features, such as Gottron papules on the metacarpophalangeal and interphalangeal joints, and heliotrope rash affecting the upper eyelids^2^. DM and PM are rare diseases with an estimated combined incidence rate of 2 per 100,000 person-years^3^. Due to the rarity of the disease, the clinical characteristics of patients with DM/PM are not well described beyond biochemical markers^4,5^ and clinical presentation^6^. Variability in clinical progression of the disease increases the difficulty of diagnosis.

Interstitial lung disease is one of the most common and potentially severe extra-muscular manifestations of DM and PM and is strongly linked to poor prognosis and early mortality^7^. ILD has been reported to occur in 21% to 80% of patients with DM or PM^8,9^, and occurs more frequently in patients with other identified risk factors, such as older age at diagnosis, the presence of anti-Jo or anti-MDA5 antibodies, arthritis/arthralgia, fever, and raised erythrocyte sedimentation rate^10^.

Management of ILD is individualised according to severity and disease subtype^11^. Because of its rarity, there have been no large randomised clinical trials that have confirmed the efficacy of treatment in patients with PM or DM who develop ILD^11^. Corticosteroids are recommended as first line treatment administered concomitantly from an early stage combined with immunosuppressive agents such as cyclosporine, tacrolimus, mycophenolate mofetil, azathioprine or cyclophosphamide, because of their corticosteroid-sparing effects and an improved treatment response^11–13^. Patients with rapidly progressive ILD or with advanced disease at presentation may require higher initial doses of corticosteroids combined with cyclophosphamide, calcineurin inhibitors, such as cyclosporine and tacrolimus, or rituximab^12^.

A meta-analysis of worldwide data published in 2020 found that the global prevalence of ILD in patients with DM or PM was 41%, but was higher in Asia (50%), and lowest in Europe (26%)^14^. Despite the frequency of this complication, the incidence rate of ILD in patients with DM or PM has not been well described. We identified only one study that reported ILD incidence rates in DM and PM; Ng et al. (2020) conducted  a population-based study in Taiwan in which the incidence rate of ILD was 1.011 per 100 person-years in patients with DM, and 0.831 per 100 person-years in PM^15^. The incidence was 125-fold higher in patients with DM and 74-fold higher in patients with PM compared to a matched healthy control group^15^.

In Japan, the annual incidence rate of DM or PM from 2004 to 2010 was estimated to be 1 to 1.3 per 100,000 person-years^16^ and the incidence rate of ILD in these patients is not known. As new treatments emerge for DM and PM, background epidemiological information will be needed to evaluate the impact of treatment on complication rates. To address this data gap and contribute to our understanding of the characteristics of patients with DM and PM and the epidemiology of ILD, we identified individuals with DM and PM and estimated the incidence rate and initial treatments for ILD using a large employment insurance database in Japan.

## Methods

### Data source

This retrospective cohort study used claims data from the Japan Medical Data Center (JMDC) database, an employment insurance database that includes around 9 million patient records of non-government employees and their family members aged below 75 years, representing between 1–2% of the entire Japanese population. The database has been operating since 2009 and captures comprehensive patient claims data (inpatient, outpatient, and pharmacy) from all healthcare services. The information includes demographic information such as age and sex, disease diagnostic codes (in International Classification of Disease 10 [ICD-10] format) and records of hospitalisation. Approximately 80% of those insured by JMDC are working adults and the remainder are their dependents. Personal identifiable information is encrypted to protect patient privacy.

### Study design, patients, and outcomes

Patients who had at least two outpatient visits and/or one inpatient admission for DM (ICD-10: M33.0 Juvenile dermatomyositis, M33.1 Other dermatomyositis and M33.9 Dermatopolymyositis, unspecified) or PM (M33.2 Polymyositis) from 01 January 2011 to 31 December 2019, were included in the study. Patients who received diagnosis codes for both DM and PM were categorized as having DM, according to the treatment guideline in Japan^13^. The first date of diagnosis in the database was defined as the index date. Eligible patients were required to have a baseline period of at least 6 months before the index date available in the database. Patients who had a diagnosis code for ILD (J84.x) at any time prior to the index date were excluded. All eligible patients were followed-up until five years after the DM/PM diagnosis, death, dis-enrolment from the database, or end of study on 31 December 2020, whichever occurred first.

Demographic characteristics, clinical characteristics, the Charlson Comorbidity Index (CCI) score^17^, cardiovascular comorbidities, and the presence of other inflammatory conditions were recorded during the 6-month baseline period. Cases of ILD (ICD-10 code J84.x—Other interstitial pulmonary diseases) that occurred after the index date were captured^18^. Cases of ILD were considered confirmed if patients had at least one inpatient hospitalisation claim or two outpatient claims for ILD.

### Statistical analysis

Descriptive statistics were used for baseline demographics and clinical characteristics. Continuous variables were summarised with mean, standard deviation (SD), median and range. Categorical variables were described with frequency and percentage. Incidence rates were calculated by dividing the number of incident ILD cases by the total number of person-years at risk. A 95% confidence interval (CI) was computed using the exact method^19^.

Cumulative risk curves were drawn to estimate and compare the rate of ILD among patients with DM versus PM over a 5-year period. Patients who did not develop ILD were censored at the time of the last claim in the database. Time to ILD incidence from DM and PM diagnosis was expressed as a median with 95% CI. The likelihood of developing ILD in patients with DM vs. PM was compared using a log-rank test.

The variables of age group (at the index date), sex, CCI, the presence of chronic obstructive pulmonary disease (COPD), cardiovascular comorbidities, inflammatory comorbidities, and rheumatoid arthritis were evaluated as risk factors using Cox proportional hazard models. All variables in the univariable analysis were included in the multivariable model after the collinearity check.

Treatments that were initiated within 3 days of the ILD diagnosis were considered first line treatments for ILD. Treatments already used prior to the ILD diagnosis were excluded from the analysis.

All analyses were conducted using SAS version 9.4 (Cary, NC, USA).

### Ethical approval and consent to participate

The database containing anonymised data was licensed by Janssen R&D from the Japan Medical Data Center Co., Ltd. All data were de-identified and fully compliant with relevant patient confidentiality requirements. According to the Japanese Ethical Guidelines for Medical and Health Research Involving Human Subjects (Ministry of Health, Labour and Welfare of Japan), individual informed consent and ethical approval were not applicable to this study. All methods were performed in accordance with the relevant guidelines and regulations.

## Results

### Study cohort

**Figure 1:**
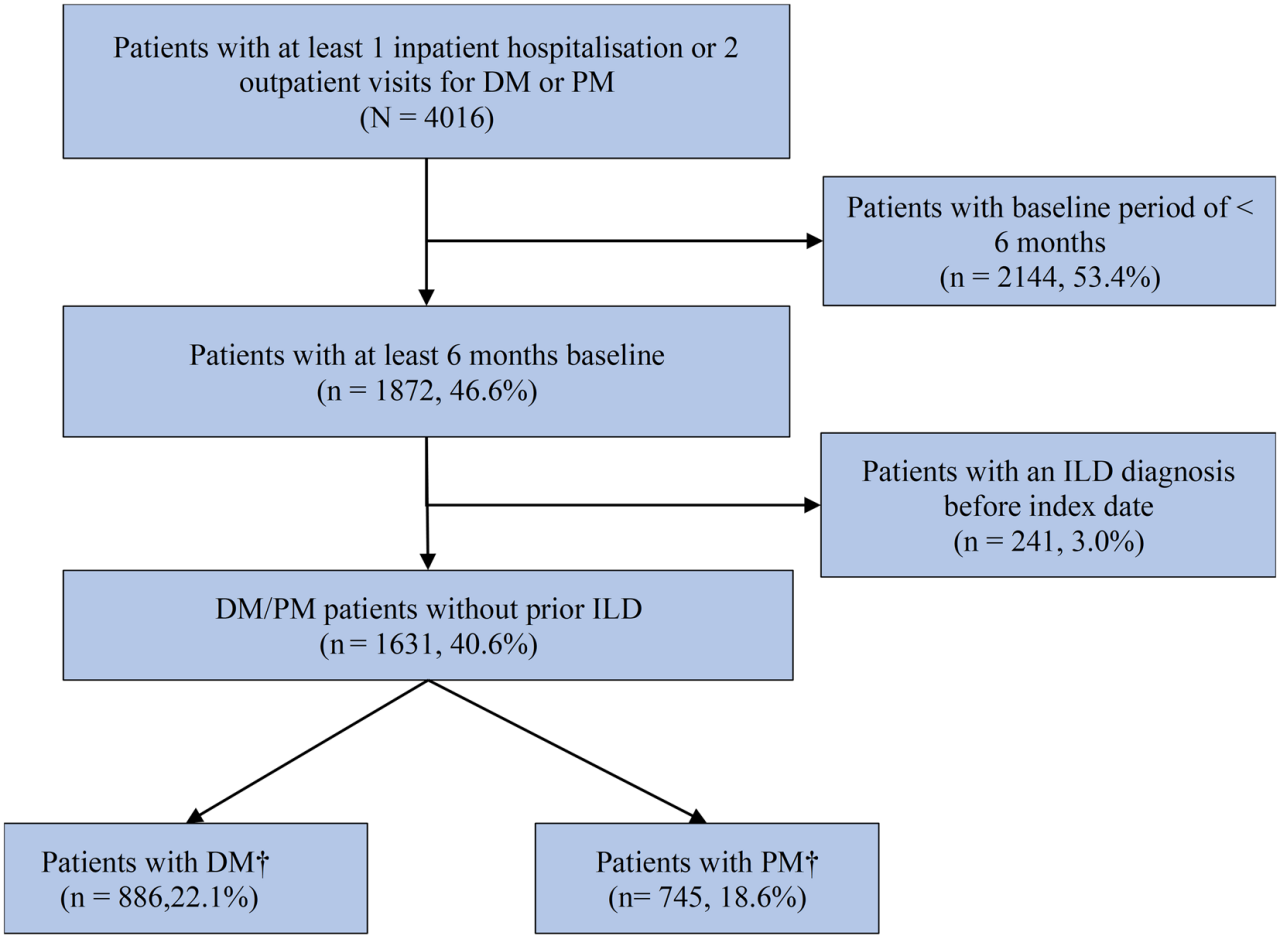
Patient flowchart. DM: dermatomyositis, PM: polymyositis, ILD: Interstitial lung disease. *The index date was the first date of identification of a DM/PM diagnosis in the database. † Patients with only one code of M33.2 during the study period were considered as having PM. Patients with codes M33.1, M33.9 or with M33.2 were grouped as having DM.

There were 4016 patients in the JMDC database with a diagnosis of DM or PM between 01 January 2011 and 31 December 2019. Of these, 2144 (53.4%) had a continuous enrolment period in the database that was shorter than 6 months preceding the index date, and 241 (3.0%) had a diagnosis of ILD before the index date, leaving 1631 patients with DM or PM without ILD at baseline who were included in the study cohort (Fig. 1). There were 886 (54.3%) patients with a diagnosis of DM (124 patients had codes for both DM and PM) and 745 (45.7%) patients with a diagnosis of PM. The mean age of patients was 46.0 years (SD 16) for DM and 49.7 years (SD 13.3) for PM (Table 1).

**Table 1:** Demographic and disease features of all eligible patients with DM and PM (01 Jan 2011 to 31 Dec 2019 in the JMDC database).

	All DM Patients (n = 886)		DM patients without ILD (n = 585)		DM patients with ILD (n = 300)		All PM Patients (n = 745)		PM patients without ILD (n = 641)		PM patients with ILD (n = 104)	
	n	%	n	%	n	%	n	%	n	%	n	%
Age at Diagnosis (years)												
Mean (SD)	46.0	16.0	44.7	16.7	48.5	14.3	49.7	13.3	49.7	13.3	49.7	13.1
Age group												
0–19	89	10.1	70	12.0	19	6.3	25	3.4	21	3.3	4	3.9
20–29	57	6.4	43	7.4	14	4.7	47	6.3	42	6.6	5	4.8
30–39	106	12.0	75	12.8	31	10.3	84	11.3	70	10.9	14	13.5
40–49	223	25.2	144	24.6	79	26.3	175	23.5	150	23.4	25	24.0
50–59	243	27.4	146	25.0	96	32.2	250	33.6	218	34.0	32	30.8
≥ 60	168	19.0	107	18.3	61	20.3	164	22.0	140	21.8	24	23.1
Sex												
Female	553	62.4	364	62.2	188	62.8	422	56.6	348	54.3	74	71.2
Male	333	37.6	221	37.8	112	37.2	323	43.4	293	45.7	30	28.9
Charlson Comorbidity Score												
Mean (SD)	0.86	1.61	0.92	1.70	0.73	1.41	0.92	1.62	0.94	1.66	0.80	1.35
Co-morbidities												
Liver disease	90	10.2	64	10.9	25	8.6	107	14.4	94	14.7	13	12.5
Chronic pulmonary disease	96	10.8	72	12.3	24	8.0	86	11.5	78	12.2	8	7.7
Peptic ulcer disease	88	9.9	54	9.2	34	11.3	76	10.2	67	10.5	9	8.7
Malignancy	51	5.8	36	6.2	15	5.0	36	4.8	33	5.2	3	2.9
Cardiovascular co-morbidities												
Cardiac arrhythmia	44	5.0	30	5.1	14	4.7	55	7.4	46	7.2	9	8.7
Congestive heart failure	43	4.9	29	5.0	14	4.7	38	5.1	35	5.5	3	2.9
Myocardial infarction	4	0.5	4	0.7	0	0.0	5	0.7	4	0.6	1	1.0
Inflammatory diseases												
Rheumatoid arthritis	86	9.7	62	10.6	24	8.0	64	8.6	45	7.0	19	18.3
Sjögren's syndrome	47	5.3	36	6.2	11	3.7	30	4.0	23	3.6	7	6.7
Other systemic involvement of connective tissue	52	5.9	35	6.0	17	5.7	42	5.6	33	5.2	9	8.7
Systemic Lupus Erythematosus	47	5.3	33	5.6	14	4.7	28	3.8	19	3.0	9	8.7
Raynaud phenomenon	29	3.3	20	3.4	9	3.0	19	2.6	17	2.7	2	1.9
Systemic sclerosis	24	2.7	14	2.4	10	3.3	11	1.5	8	1.3	3	2.9
Psoriasis/Psoriatic arthritis	16	1.8	9	1.5	7	2.3	4	0.5	4	0.6	0	0.0

DM, dermatomyositis, PM, polymyositis; SD: Standard deviation.

*Four most common frequently reported co-morbidities in the CCI (Supplementary Table 1).

^Includes other overlap syndromes, Behçet disease, polymyalgia rheumatica diffuse (eosinophilic) fasciitis, multifocal fibrosclerosis, elapsing panniculitis, hypermobility syndrome and systemic disorders of connective tissue in diseases in neoplastic disease and hypersensitivity disorders.

Females comprised 62.4% of patients with DM and 56.6% of patients with PM. 

The mean CCI score was 0.86 (SD 1.61) in patients with DM, and 0.92 (SD 1.62) in patients with PM; 80.6% of patients with DM and 78.2% with PM had CCI scores of 0 and 1 (Supplementary Table 1). Co-morbidities prior to the index date were distributed similarly in patients with DM or PM. The most frequently observed co-morbidities were liver disease (10.2% in DM, 14.4% in PM), chronic pulmonary disease (excluding ICD-10 code J84.x) (10.8% in DM, 11.5% in PM), rheumatic arthritis (9.7% in DM and 8.6% in PM), and peptic ulcer disease (9.9% in DM, 10.2% in PM).

### Incidence rate of ILD

**Figure 2:**
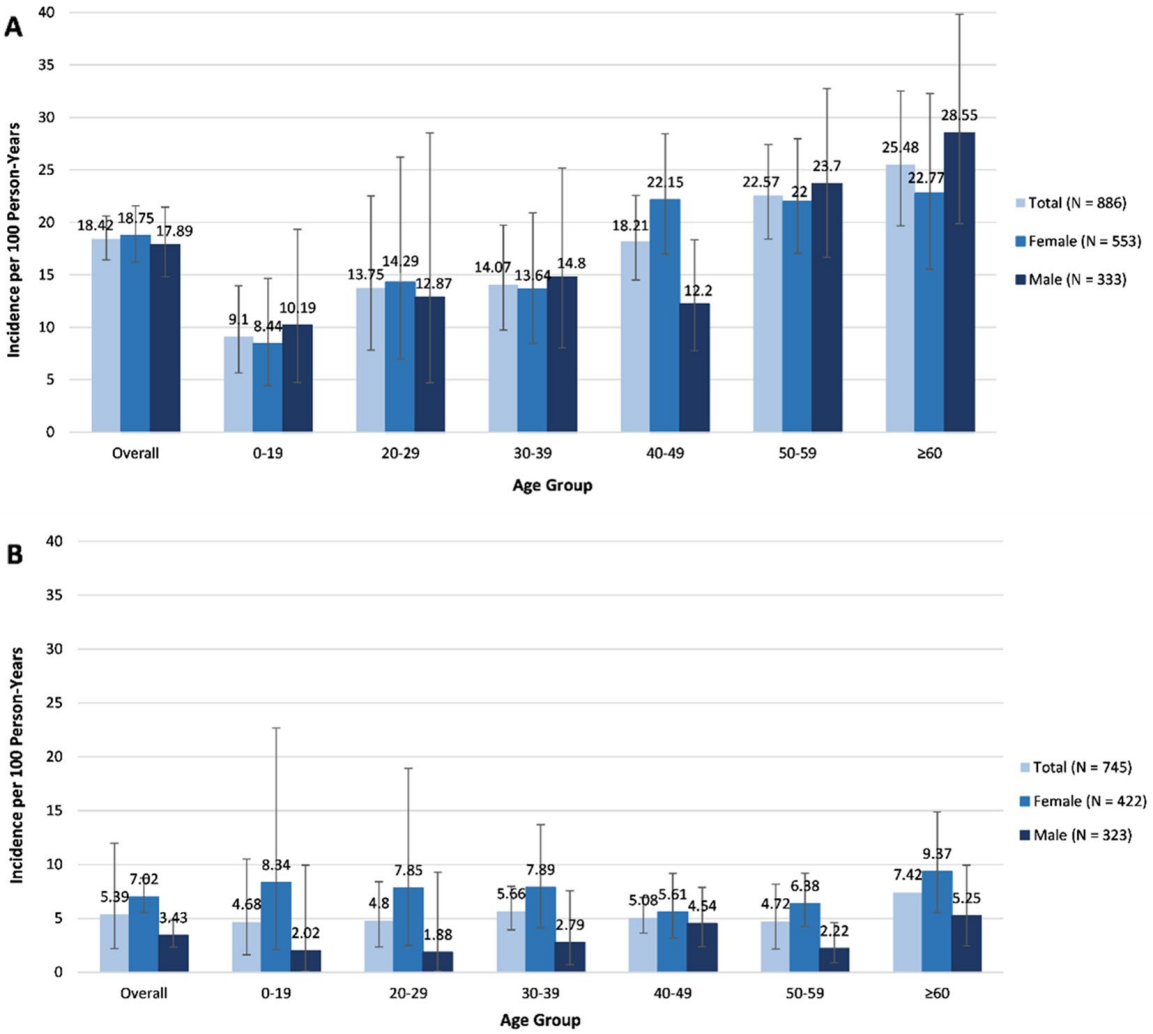
Sex-specific and age-specific incidence of ILD in patients with A) DM B) PM (per 100 persons-years). DM: dermatomyositis, ILD: Interstitial lung disease; PM: polymyositis; error bars: 95% confidence interval. Tabulated data are provided in Supplement Table 4.

There were 300 (34%) patients with DM and 104 (14%) patients with PM who developed ILD during the follow-up period. The incidence rate of ILD (per 100 person-years) was 18.42 (95% CI 16.42–20.59) in patients with DM (Fig. 2A), and 5.39 (95% CI 4.43–6.5) in patients with PM (Fig. 2B).

In patients with DM, the incidence rate of ILD in patients with DM increased with age, with the largest incremental increase (62%) between 30–39 years and 40–49 years of age in females, and between 40–49 and 50–59 years of age (94% increase) in males (Fig. 2A).

In patients with PM, the overall incidence rate of ILD was higher in females (7.02 per 100 person-years, 95% CI 5.55–8.76), than males (3.43 per 100 person-years, 95% CI 2.35–4.83). In view of the higher incidence rate of DM and PM that we observed compared to that reported by Ng et al. 2020^15^ in Taiwan, we conducted a sensitivity analysis applying the same exclusion criteria used by Ng et al.; excluding DM patients with a co-diagnosis of PM, and all patients with any systemic autoimmune rheumatic disease (rheumatoid arthritis, Sjögren’s syndrome, SLE, and systemic sclerosis). The resulting incidence rate of ILD was 16.72 (95% CI 14.53–19.15) in patients with DM and 4.57 (95% CI 3.64–5.68) in patients with PM, which did not differ greatly from the original results (Supplementary Table 2).

### Risk of ILD in patients with DM compared with PM

**Figure 3:**
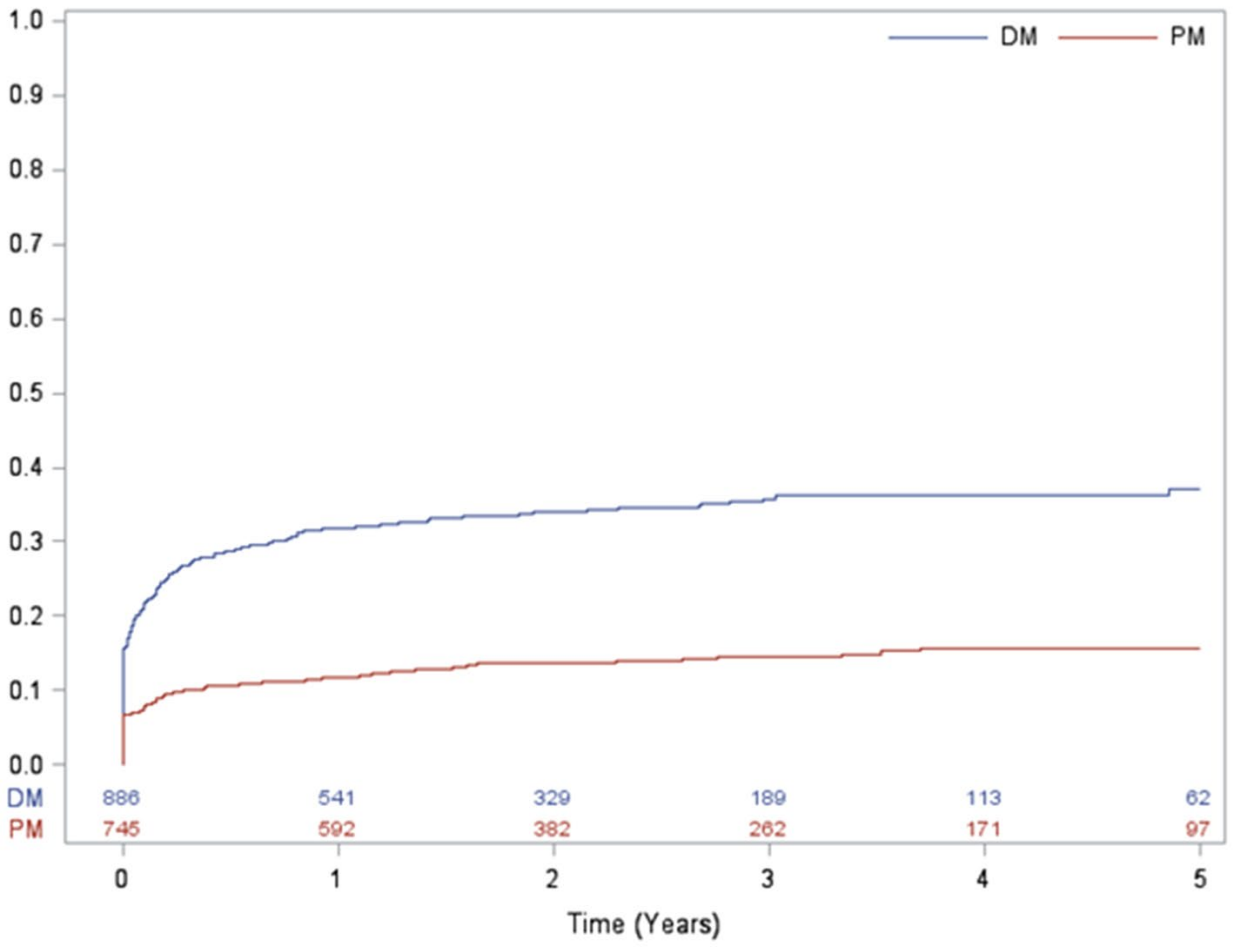
Cumulative risk curves for ILD in patients with DM and PM.

In the crude analysis of risk of ILD, patients with DM were at higher risk of developing ILD as compared to patients with PM (HR 2.745, 95% CI 2.19–3.43) (Table 2). After adjusting for confounders of sex, age-group (≥ 60 and < 60) and CCI score, the risk of ILD in patients with DM remained significantly higher than in patients with PM (HR 2.72, 95% CI 2.18–3.41). The cumulative incidence curve showed similar findings with log-rank test p < 0.001 for the risk of ILD in patients with DM compared with PM. The rate of ILD diverged markedly between treatment groups during the first year of follow-up, reaching 31.9% at year 1 for DM and 11.8% for PM (Fig. 3).

**Table 2:** Risk of ILD in patients with DM compared with patients with PM using a Cox regression model (n = 1631).

Diagnosis	No. of patients	No. of ILD events	Person-years	Crude HR (95% CI)	*p* value	Adjusted* HR (95% CI)	*p* value
PM	745	104	1783.69	Reference		Reference	
DM	886	300	1521.44	2.745 (2.196, 3.433)	< 0.0001	2.726 (2.179, 3.410)	< 0.0001

*p*-value: chi-square test comparing patients with versus without ILD after the index date. The index date was the first date of identification of a DM or PM diagnosis in the database.

CI: confidence interval; DM: dermatomyositis HR: hazard ratio; ILD: interstitial lung disease; PM: polymyositis.

*Adjusted for sex, age (≥ 60; < 60), and CCI score (≥ 2; 1; 0).

### Risk factors for ILD

The risk factors identified for ILD were different in patients with DM versus PM. For patients with DM, the univariable analysis demonstrated that age and CCI were factors associated with ILD risk. Only older age was identified as a significant risk factor in the multivariable model (compared to 0–39 year-olds, HR 1.529, 95% CI 1.099–2.128 for 40–49 year-olds; HR 1.779, 95% CI 1.293–2.449 for 50–59 year-olds; and HR 1.722, 95% CI 1.202–2.466 for ≥ 60 year-olds). For patients with PM, being female and having inflammatory comorbidities or rheumatoid arthritis were risk factors for ILD in the univariable analysis. Being female (HR 1.935, 95% CI 1.259–2.972) and rheumatoid arthritis (HR 2.924, 95% CI 1.265–6.760) remained significant in the multivariable analysis (Table 3).

**Table 3:** Univariable and multivariable models* for risk factors for ILD in patients with DM and PM.

	Patients with DM				Patients with PM			
	Univariable		Multivariable		Univariable		Multivariable	
	HR (95% CI)	*p*	HR (95% CI)	*p*	HR (95% CI)	*p*	HR (95% CI)	*p*
Age group								
0–39	Reference		Reference		Reference		Reference	
40–49	1.491 (1.073–2.074)	0.0175	1.529 (1.099–2.128)	0.0118	0.959 (0.545–1.690)	0.8858	0.950 (0.538–1.678)	0.8593
50–59	1.681 (1.225–2.306)	0.0013	1.779 (1.293–2.449)	0.0004	0.865 (0.506–1.478)	0.5957	0.776 (0.448–1.343)	0.3641
≥ 60	1.624 (1.143–2.307)	0.0068	1.722 (1.202–2.466)	0.0030	1.049 (0.592–1.859)	0.8706	1.124 (0.617–2.042)	0.7033
Sex								
Female	1.018 (0.805–1.286)	0.8840	1.023 (0.805–1.300)	0.8505	1.965 (1.285–3.003)	0.0018	1.935 (1.259–2.972)	0.0026
Male	Reference		Reference		Reference		Reference	
CCI								
0	Reference		Reference		Reference		Reference	
1	0.988 (0.744–1.312)	0.9346	1.017 (0.739–1.401)	0.9165	1.539 (0.992–2.387)	0.0542	1.422 (0.842–2.402)	0.1876
≥ 2	0.721 (0.522–0.994)	0.0462	0.711 (0.483–1.048)	0.0848	0.846 (0.492–1.457)	0.5471	0.719 (0.361–1.430)	0.3472
COPD								
0	Reference		Reference		Reference		Reference	
1	0.670 (0.442–1.017)	0.0600	0.731 (0.462–1.157)	0.1807	0.637 (0.310–1.311)	0.2206	0.556 (0.258–1.195)	0.1325
Cardiovascular comorbidities								
0	Reference		Reference	0.8303	Reference		Reference	
1	0.886 (0.584–1.345)	0.5700	1.051 (0.667–1.655)		0.888 (0.463–1.704)	0.7210	0.931 (0.464–1.869)	0.8405
Inflammatory comorbidities								
0	Reference		Reference	0.4528	Reference		Reference	
1	0.926 (0.697–1.231)	0.5983	1.155 (0.793–1.680)		1.794 (1.157–2.781)	0.0090	0.923 (0.426–1.999)	0.8385
Rheumatoid arthritis								
0	Reference		Reference	0.2470	Reference		Reference	
1	0.785 (0.517–1.191)	0.2547	0.734 (0.435–1.239)		2.479 (1.508–4.077)	0.0003	2.924 (1.265–6.760)	0.0121

CCI, Charlson comorbidity index, CI: confidence interval, DM: dermatomyositis, ILD: interstitial lung disease, PM: polymyositis: COPD: chronic obstructive pulmonary disease.

*Including all variables.

### Treatment pattern for ILD

Of 404 patients who developed ILD, 82 (20.3%) initiated a new drug treatment within 3 days after the ILD diagnosis and 40.0% initiated a drug treatment that they had received previously. Glucocorticoids with or without immunosuppressive drugs were the most frequently newly prescribed treatments, used by 86.6% (71/82) of patients. The most frequently newly prescribed immunosuppressive drug was tacrolimus, prescribed for 47.6% (39/82) of patients. We also explored a 7-day window after the ILD diagnosis and observed a similar prescription pattern (Supplementary Table 3).

## Discussion

Study of DM and PM is difficult because of the rarity of these diseases, making it challenging to identify cohorts large enough to provide meaningful data. We used a large employment database in Japan and identified 1631 patients with a diagnosis of DM or PM over the almost 9-year study period. We observed an incidence rate of ILD of 18.42 (95% CI 16.42–20.59) per 100 person-years in patients with DM, and 5.39 (95% I 4.43–6.5) per 100 person-years in patients with PM. The incidence rate of ILD increased markedly between the fourth and fifth decades in women, and between the fifth and sixth decades in men, suggesting that close monitoring for ILD in these patients during these years could be warranted.

The rates we observed are approximately tenfold higher than those observed in the only other study of ILD in patients with DM or PM by Ng et al., 2020 in Taiwan^15^. Like Ng et al., we observed a higher ILD incidence in patients with DM than with PM, even after adjusting for underlying differences between the two groups. We also observed higher incidence rates of ILD in women than in men with PM, but consistent associations between age and ILD risk observed by Ng et al., were not replicated in our study. In the Taiwan study only 5.5% of patients with DM and 4.6% with PM developed ILD over the follow-up period, compared with 24.8% of patients with DM/PM in our study, which is more consistent with reported estimates ILD occurring in at least 21%, and up to 80% of patients with DM/PM^8,9^. In discussing the low proportion of ILD in patients with PM and DM in their report, Ng et al., listed differences due to ethnicity and a strict ILD definition as potential contributors.

In contrast to the approach used by Ng et al. to define the DM/PM cohorts, we did not exclude patients with concurrent inflammatory disorders. However, a sensitivity analysis showed that exclusion of these patients had little impact on the incidence estimates, suggesting a minor role of differences in inclusion criteria in explaining the differences between the two studies. Ng et al. used a stringent case definition of ICD-9 codes of 515 Post-inflammatory pulmonary fibrosis, 516.3 Idiopathic interstitial pneumonia, 516.8 Other specified alveolar and parietoalveolar pneumonopathies, 516.9 Unspecified alveolar and parietoalveolar pneumonopathy and 517 Lung involvement in conditions classified elsewhere. In contrast, the J84.x codes in ICD-10 encompass numerous conditions not covered by the ICD-9 codes employed by Ng et al.^18^, which likely allowed us to capture more cases of ILD. In addition to a more restricted set of codes, Ng et al., 2020 also required evidence of radiological or pathological confirmation of ILD, further reducing the sensitivity of the ILD case definition in the study by Ng et al.

Finally, ethnic differences may also have played some role in the observed differences between the study results. ILD has been more commonly reported as an adverse event in drug safety databases in Japan as compared to rest of the world^20^, possibly due to enhanced pharmaco-ethnic susceptibility of the Japanese population to ILD^21,22^. ILD incidence rates from other countries with which to compare the data from Taiwan and Japan are not currently available. We therefore conclude that our study is not directly comparable with that of Ng et al., primarily due to a less stringent ILD case definition, and a possible contribution of ethnic differences in susceptibility to ILD.

Corticosteroids and immunosuppressive agents are considered first line therapy for ILD, although no evidence-based guidelines exist due to the rarity of the disease which has hindered conduct of randomised, controlled trials^11,23^. We observed that glucocorticoids and/or immunosuppressive drugs, mainly tacrolimus, were the most frequent newly prescribed drugs initiated early after the ILD diagnosis. Tacrolimus is recommended for progressive or refractory ILD in which conventional treatments such as cyclosporin have no efficacy, and for patients with anti–aminoacyl-tRNA synthetase autoantibodies^11,12^.

Strengths of our study included the use of a large, longitudinal claims database that allowed identification of a large cohort of patients with DM/PM and subsequent follow-up for ILD for up to 9.5 years. The sex distribution observed over the study period in JMDC was similar to the sex distribution of Japan^24^, suggesting that the study findings are representative of the working population in Japan.

Potential limitations of the study relate to the JMDC database which only includes employed individuals and their families until age 75 years. Individuals who cannot work are not covered under JMDC insurance, and patients with the most severe disease/complications may not be captured. In our study, employees contributed to 56.1% of the total cohort, and these patients may have had less severe disease than persons unable to work. Furthermore, given that age is a risk factor for ILD in patients with DM, the absence of data in adults older than 75 years may mean that the incidence is underestimated in our study. We used prescriptions of new drugs within 3 days after the ILD diagnosis as an indicator of first line treatment for ILD. Only 20.3% of patients initiated a new treatment in this timeframe and we are unable to determine the relationship between the prescription and the ILD diagnosis in the 40.0% of patients who received a previously prescribed medication. We cannot exclude that patients may have been given a diagnosis in order to receive medical reimbursement for services examinations in Japan. For example, methotrexate is reimbursed for rheumatoid arthritis but not DM/PM treatment in Japan, and it is feasible that some patients may have been given a diagnosis of rheumatoid arthritis in order to have their treatment reimbursed. Confirmatory findings from electromyography, muscle biopsy, muscle MRI, skin biopsy, and creatine kinase levels were not available to us. On the other hand, the prescription of drugs for treatment of the diagnosis are less likely to be biased. Finally, the database lacks clinical information to allow diagnostic confirmation, assessment of disease severity, or clinical decision-making underlying drug prescribing patterns.

In conclusion, ILD is a common complication of DM/PM with an incidence rate in our study of 18.42 (95% CI 16.42–0.59) per 100 person-years in patients with DM and 5.39 (95% CI 4.43–6.5) in patients with PM. The incidence rate of ILD was higher in women than in men in PM, and more than twofold higher in patients with DM than with PM. Additional studies conducted in other countries using comparable case definitions are needed to understand the epidemiology of ILD and the potential contribution of co-existing inflammatory conditions, such as rheumatoid arthritis, to risk. Such data will be needed to measure the impact of new treatments for DM/PM. Studies using large databases that accumulate longitudinal real-world data can be used to support our understanding of the epidemiology and potential complications of rare diseases.

### Supplementary Information

Supplementary Tables.

## Data Availability

The data underlying this article were provided by the JMDC under license. Data will be shared on request to the corresponding author with permission of JMDC.

## Abbreviations

CCI: Charlson comorbidity index
CI: Confidence interval
COPD: Chronic obstructive pulmonary disease
DM: Dermatomyositis
HR: Hazard ratio
ICD: International classification of disease
PM: Polymyositis
ILD: Interstitial lung disease
JMDC: Japan medical data center
SD: Standard deviation
