# Supplementary material for: Characteristics and risk of interstitial lung disease in dermatomyositis and polymyositis: a retrospective cohort study in Japan
Source: Sci Rep. 2023 Oct 11;13:17172. doi: 10.1038/s41598-023-44092-9 (PMC10567809; doi:10.1038/s41598-023-44092-9)
Supplement: Supplementary file 1 — Supplementary Tables. [file 41598_2023_44092_MOESM1_ESM.docx]

**Characteristics and risk of interstitial lung disease in dermatomyositis and polymyositis: a retrospective cohort study in Japan**

Qingqing Hu, Kuan-Chih Huang, Choo Hua Goh, Yumi Tsuchiya, Yanfang Liu, Hong Qiu

**SUPPLEMENT**

**Supplementary Table 1 Charlson comorbidity score and components in patients with DM, PM, and concurrent DM and PM**

|  | **All patients with DM** | | **All patients with PM** | |
| --- | --- | --- | --- | --- |
|  | **(n = 886)** | | **(n =745)** | |
|  | **n** | **%** | **n** | **%** |
| **Charlson Comorbidity Score** |  |  |  |  |
| 0 | 532 | 60.1 | 429 | 57.6 |
| 1 | 182 | 20.5 | 161 | 20.6 |
| 2 | 90 | 10.2 | 74 | 9.9 |
| 3 | 36 | 4.1 | 32 | 4.3 |
| 4+ | 46 | 5.2 | 49 | 6.6 |
| **Charlson Comorbidity** |  |  |  |  |
| Mild liver disease | 90 | 10.2 | 107 | 14.4 |
| Rheumatic disease | 109 | 12.3 | 81 | 10.9 |
| Chronic pulmonary disease | 96 | 10.8 | 86 | 11.5 |
| Peptic ulcer disease | 88 | 9.9 | 76 | 10.2 |
| Any malignancy, including lymphoma and leukemia, except malignant neoplasm of skin | 49 | 5.5 | 33 | 4.4 |
| Congestive heart failure | 43 | 4.9 | 38 | 5.1 |
| Peripheral vascular disease | 38 | 4.3 | 38 | 5.1 |
| Cerebrovascular disease | 25 | 2.8 | 31 | 4.2 |
| Diabetes without chronic complication | 19 | 2.1 | 22 | 3 |
| Diabetes with chronic complication | 17 | 1.9 | 17 | 2.3 |
| Metastatic solid tumor | 15 | 1.7 | 9 | 1.2 |
| Renal disease | 6 | 0.7 | 11 | 1.5 |
| Myocardial infarction | 4 | 0.5 | 5 | 0.7 |
| Hemiplegia or paraplegia | 2 | 0.2 | 5 | 0.7 |
| Dementia | 2 | 0.2 | 3 | 0.4 |
| Moderate or severe liver disease | 1 | 0.1 | 2 | 0.3 |
| AIDS/HIV | 1 | 0.1 | 1 | 0.1 |

AIDS: acquired immunodeficiency syndrome; HIV: human immunodeficiency virus; DM: dermatomyositis; PM: polymyositis.

**Supplementary Table 2 Sex-specific incidence of ILD among patients with DM and PM without rheumatoid arthritis, systemic sclerosis, systemic lupus erythematosus or Sjogren's syndrome (per 100 person-years)**

|  | **Total** | | | | **Female** | | | | **Male** | | | |
| --- | --- | --- | --- | --- | --- | --- | --- | --- | --- | --- | --- | --- |
|  | No. of patients | No. of ILD events | Person-years | Incidence  (95% CI) | No. of patients | No. of ILD events | Person-years | Incidence  (95% CI) | No. of patients | No. of ILD events | Person-years | Incidence  (95% CI) |
| **DM** | 635 | 202 | 1207.92 | 16.72  (14.53, 19.15) | 389 | 129 | 700.66 | 18.41  (15.43, 21.80) | 246 | 73 | 507.26 | 14.39  (11.36, 17.99) |
| **PM** | 648 | 78 | 1705.93 | 4.57  (3.64, 5.68) | 350 | 50 | 891.76 | 5.61  (4.21, 7.33) | 298 | 28 | 814.17 | 3.44  (2.33, 4.90) |

CI, confidence interval; DM: dermatomyositis; PM: polymyositis, ILD: interstitial lung disease

**Supplementary Table 3 New treatments initiated with 3 days or 7 days* after the ILD in patients with DM and PM**

|  |  | **Within 3 days from ILD diagnosis date** | | **Within 7 days from ILD diagnosis date** | |
| --- | --- | --- | --- | --- | --- |
| **Drug category** | **Drug name** | **N=82** | **%** | **N=103** | **%** |
| Glucocorticoid | Prednisolone | 16 | 19.51 | 22 | 21.36 |
|  | Methylprednisolone | 6 | 7.32 | 3 | 2.91 |
|  | Dexamethasone | 3 | 3.66 | 3 | 2.91 |
|  | Prednisolone + methylprednisolone | 4 | 4.88 | 11 | 10.68 |
|  | Prednisolone + methylprednisolone + dexamethasone | 1 | 1.22 | 1 | 0.97 |
| Immunosuppressive | Methotrexate | 4 | 4.88 | 4 | 3.88 |
|  | Tacrolimus | 6 | 7.32 | 6 | 5.83 |
|  | Cyclosporin A | 2 | 2.44 | 1 | 0.97 |
|  | Azathioprine | 2 | 2.44 | 2 | 1.94 |
|  | Methotrexate + tacrolimus | 1 | 1.22 | 1 | 0.97 |
| Glucocorticoid + immunosuppressive | Prednisolone + tacrolimus | 13 | 15.85 | 17 | 16.5 |
|  | Methylprednisolone + tacrolimus | 10 | 12.2 | 6 | 5.83 |
|  | Prednisolone + methylprednisolone + tacrolimus | 6 | 7.32 | 12 | 11.65 |
|  | Prednisolone + cyclosporin | 1 | 1.22 | 3 | 2.91 |
|  | Prednisolone + azathioprine + tacrolimus | 3 | 3.66 | 3 | 2.91 |
|  | Cyclosporin + methylprednisolone | 0 | 0 | 3 | 2.91 |
|  | Prednisolone + cyclosporin + methylprednisolone | 1 | 1.22 | 1 | 0.97 |
|  | Prednisolone + mycophenolic acid | 1 | 1.22 | 1 | 0.97 |
| Plasmapheresis | Plasmapheresis | 1 | 1.22 | 1 | 0.97 |
| Glucocorticoid + immunosuppressive + immunoglobulin | Methylprednisolone + cyclosporin + immunoglobulins | 1 | 1.22 | 0 | 0 |
|  | Cyclosporin + immunoglobulins + methylprednisolone + prednisolone | 0 | 0 | 1 | 0.97 |
|  | Methylprednisolone + tacrolimus + immunoglobulins | 0 | 0 | 1 | 0.97 |

CI: confidence interval, DM: dermatomyositis, HR: hazard ratio, ILD: Interstitial lung disease, PM: polymyositis

*Note that some patients may have commenced a new drug between day 3 and day 7. This results in a new drug combination that is different from the original day 3 drug category.

**Supplementary Table 4 Sex-specific and age-specific incidence of ILD among PM patients (per 100 persons-year)**

| Age, years | **Total (N = 745)** | | | | |  | **Female (N = 422)** | | | | |  | **Male (N = 323)** | | | | |
| --- | --- | --- | --- | --- | --- | --- | --- | --- | --- | --- | --- | --- | --- | --- | --- | --- | --- |
|  | No. patients | No. of ILD events | Person-years | Incidence | 95% CI |  | No. of patients | No. of ILD events | Person-years | Incidence | 95% CI |  | No. of patients | No. of ILD events | Person-years | Incidence | 95% CI |
| Total | 745 | 104 | 1930.20 | 5.39 | (4.43, 6.50) |  | 422 | 74 | 1054.50 | 7.02 | (5.55, 8.76) |  | 323 | 30 | 875.70 | 3.43 | (2.35, 4.83) |
| 0-19 | 25 | 4 | 85.53 | 4.68 | (1.49, 11.28) |  | 11 | 3 | 35.99 | 8.34 | (2.12, 22.69) |  | 14 | 1 | 49.54 | 2.02 | (0.10, 9.96) |
| 20-29 | 47 | 5 | 104.14 | 4.80 | (1.76, 10.64) |  | 26 | 4 | 50.98 | 7.85 | (2.49, 18.93) |  | 21 | 1 | 53.17 | 1.88 | (0.09, 9.28) |
| 30-39 | 84 | 14 | 247.14 | 5.66 | (3.22, 9.28) |  | 52 | 11 | 139.47 | 7.89 | (4.15, 13.71) |  | 32 | 3 | 107.67 | 2.79 | (0.71, 7.58) |
| 40-49 | 175 | 25 | 491.70 | 5.08 | (3.36, 7.40) |  | 95 | 14 | 249.49 | 5.61 | (3.19, 9.19) |  | 80 | 11 | 242.21 | 4.54 | (2.39, 7.89) |
| 50-59 | 250 | 32 | 678.42 | 4.72 | (3.28, 6.58) |  | 152 | 26 | 407.77 | 6.38 | (4.25, 9.21) |  | 98 | 6 | 270.65 | 2.22 | (0.90, 4.61) |
| ≥60 | 164 | 24 | 323.27 | 7.42 | (4.87, 10.88) |  | 86 | 16 | 170.80 | 9.37 | (5.55, 14.89) |  | 78 | 8 | 152.47 | 5.25 | (2.44, 9.96) |
